# Reflections on a Health Psychology Service for Patients with Uveal Melanoma: The Challenge of Psychological Screening and Intervention When Distress is ‘Normal’

**DOI:** 10.1007/s10880-018-9595-2

**Published:** 2018-11-21

**Authors:** Laura Hope-Stone, Janice Ablett, Peter Salmon

**Affiliations:** 1 Liverpool Ocular Oncology Centre & Clinical Health Psychology Service-Cancer, Royal Liverpool and Broadgreen University Hospital, NHS Trust, Prescot St, Liverpool, L7 8XP UK; 2 Institute of Psychology, Health & Society, University of Liverpool, Whelan Building, Brownlow Hill, Liverpool, L69 3GB UK

**Keywords:** Cancer, Assessment, Screening, Distress, Coping, Intervention

## Abstract

We appraise the role of screening for distress as part of health psychology assessment of patients newly diagnosed with cancer. We reviewed records of consecutive patients who accepted a health psychologist’s assessment over 4 years, examining convergence and divergence of the result of screening (whether patients reached threshold as ‘cases’) with the psychologist’s clinical judgment of need for intervention. Of 261 patients, 88 (33.7%) were ‘cases’. Of these, need for psychological intervention was identified in 70 (79.5%). Of the 173 (66.3%) ‘non-cases’, need was identified in 59 (34.1%). Examination of cases where the psychologist’s judgment diverged from screening showed that ‘caseness’ can arise from distress that patients can manage themselves and, conversely, that psychological needs arise in the absence of overt distress. Formal screening may not identify need for psychological intervention. The psychologist’s role is to make expert judgments of patients’ current and future needs. Dialogue with patients should be the vehicle for assessment.

In 2004, Liverpool Ocular Oncology Centre (LOOC) established an innovative post for a health psychologist to offer psychological assessment and early intervention to every patient with uveal melanoma. The service remains internationally unique in its application of health psychology to this population, as far as we know. Nevertheless, lessons from it are potentially relevant for psychologists more broadly, particularly those working with other populations around the time of life threatening or life-changing diagnoses. Here, we consider the value of a health psychology assessment for patients recently diagnosed with cancer.

Uveal melanoma is a rare cancer, with around 500 new cases diagnosed annually in the UK. LOOC is the largest of four tertiary centres for uveal melanoma in the UK. Patients attend from across the country, being seen within 4 weeks of referral. Most are referred by their local ophthalmologist, having presented with visual disturbances. However, around a third are referred after routine eye tests and are asymptomatic (Damato, 2001, 2010). Most patients with uveal melanoma are treated 3–8 days after diagnosis. Whilst the primary tumour can be treated effectively, nearly half the patients will develop liver metastases for which there is no proven curative treatment (Kujala, Mäkitie, & Kivelä, 2003). Face to face contact with LOOC staff is brief for most patients who receive a cancer diagnosis. They are treated within 1 or 2 days of their first appointment, being discharged soon after. Although most patients are followed up at LOOC after 6 months, others are discharged to their local hospital. While the clinical staff have a crucial supportive role (Lilliehorn, Hamberg, Kero, & Salander, 2010), their necessarily limited contact with patients, given the pace at which diagnosis and treatment proceeds, constrains this. Therefore, LOOC established the Health Psychology Service to ensure that psychological needs were not neglected during this intense focus on clinical care. The Health Psychologist (HP) is a full member of the Ocular Oncology Multi-Disciplinary Team (MDT), routinely attending the weekly MDT meeting where she can discuss concerns with clinical staff about individual patients and offer support and advice to the team. However, her main role is to provide assessment and appropriate intervention directly to patients.

The HP’s assessment is designed to determine whether patients have psychological needs that warrant her specific intervention, interpreted broadly to include emotional distress that requires intervention at the time, psychological needs that might not manifest as overt distress and adjustment difficulties that might lead to future needs without early intervention. Because many patients live far away, psychological intervention is mostly by telephone (Watson et al., 2013; Watson, White, Lynch, & Mohammed, 2017). Intervention is based on a collaborative approach which provides a supportive context for emotional disclosure. The HP elicits patients’ concerns, validates normal responses, facilitates effective coping strategies and provides structured help with anxiety and worry management. The HP also needs to decide whether to refer patients to other agencies for more complex or persistent needs than she can address. Patients can, of course, accept or decline the HP’s intervention or referral; she would only over-ride this if she judged that a patient was at risk of harm, or harming others.

Psychological screening has become central to policy internationally to ensure that psychological need is recognized, assessed and addressed in patients with cancer (Brennan, 2012; Deshields & Nanna, 2010). However, its value and utility has been challenged on several grounds (Coyne, 2013a; Salmon, Clark, McGrath, & Fisher, 2015): distress is ‘normal’ after a traumatic diagnosis; many patients who are ‘distressed’, in that they reach a predetermined threshold on a screening questionnaire, do not want psychological help (Coyne, 2013b); and routine psychological intervention in the context of normal emotional reactions to challenges can be detrimental (Litz, Gray, Bryant, & Adler, 2002). Furthermore, measures of distress in the absence of a psychological formulation do not indicate the specific intervention needed (Deshields & Nanna, 2010).

In our service, we screen, but do not make psychological intervention contingent on this. Our policy is that, even in the absence of formal indicators of distress, the potentially traumatic and life-changing experience of uveal melanoma entitles any patient to the opportunity to meet the HP. Around 70% of patients are admitted for in-patient treatment and are seen in person by the HP following surgery. If the patient agrees, the HP conducts an assessment and discusses the patient’s psychological needs with him/her. The assessment takes the form of a conversational interview and, as recommended by Bonacchi et al. (2010), includes a formal measure of distress: the Hospital Anxiety and Depression Scale (HADS; Zigmond & Snaith, 1983), which is widely used to screen cancer populations (Vordermaier & Millman, 2011).

The HP’s assessment and formulation is guided by the ‘Stress and Coping Model’ (Lazarus & Folkman, 1984). This postulates an ‘appraisal’ process whereby individuals evaluate the personal significance of challenges such as those in cancer diagnosis and treatment. Based on this appraisal, the model assumes that patients develop coping strategies which reflect their available personal, social and psychological resources. The HP therefore ‘interprets’ the HADS score according to broader information about these elements of the model, gained from dialogue with the patient. Crucially, the HP needs to decide whether patients are likely to recover without her intervention, so that she does not impair their inherent coping ability (Brennan, 2004; Dekker et al., 2017; Litz et al., 2002) No algorithm exists to integrate this information, so the HP needs to make expert clinical judgments based on formulating patients’ needs. Decisions as to whether patients receive follow-up or referral to other services arise from discussing her judgment with patients themselves. Our approach therefore allows us to examine what a formal screen for distress tells us in the context of the broader assessment.

Based on experience in other cancer settings, and on the broader literature on screening for distress in cancer care, we anticipated that there would be a clinically significant proportion of patients whose psychological need diverged from the screening tool (Merckaert et al., 2010; Söllner, Maislinger, König, DeVries, & Lukas, 2004). However, we were unsure as to the size of this proportion in patients newly diagnosed with uveal melanoma. In the present study, we therefore sought (i) to determine to what extent HADS screening reflected patients’ psychological needs as identified by the HP and (ii) to identify reasons for any divergence of the HP’s judgment from the result of screening.

## Methods

This study was approved as a service evaluation by the Royal Liverpool & Broadgreen University Hospital Trust Clinical Effectiveness Department (TA 1840) and was performed in accordance with ethical standards as specified in the 1964 Declaration of Helsinki. We reviewed clinical records of all patients who accepted a routine health psychology assessment over 4 years (April 2014–March 2018), noting also the final decision recorded about whether the HP thought that post-discharge psychological intervention was necessary and the score on the HADS administered during the assessment. We categorized patients according to probable clinical ‘caseness’, using a criterion of ≥ 8 (Vodermaier & Millman, 2011) on either HADS subscale (anxiety or depression). We noted demographic and clinical variables from patients’ clinical records. Variables used in statistical analyses included gender, age (categorized by a median split) and treatment (enucleation vs. conservative treatments).

To determine the extent of convergence and divergence between the two ways of categorizing patients, we crosstabulated HADS caseness with the HP’s clinical judgment as to whether the patient needed psychological intervention using *χ*^2^ to test the significance of the relationship (significance criterion *p* < .05). We then used *χ*^2^ to explore whether HADS caseness and the HP’s judgment of psychological need were related to gender, age or treatment. Finally, we used log linear analysis to explore the 3-way interactions of HADS caseness and the HP’s judgment with each of gender, age or treatment; that is, to show whether these variables were related to the convergence or divergence between caseness and the HP’s judgment. After checking for conformity with assumptions, we fitted a saturated log linear model, examining evidence of 3-way and higher order effects before proceeding to test individual effects. We report Pearson *χ*^2^ throughout the results (likelihood ratio *χ*^2^ produced similar results). To protect against Type 1 errors in the exploratory analyses, our significance criterion for these was *p* < .01. We used SPSS v24 for statistical analyses.

To identify reasons for convergence and divergence we reviewed the HP’s clinical records. To illustrate the reasons we created case vignettes based on assessments of individual patients falling into each of the four cells resulting from cross-tabulation of HADS caseness with the HP’s judgment. We categorized these as ‘true’ or ‘false’ ‘positives’ or ‘negatives’, regarding the HP’s judgment as the ‘gold standard’. To preserve anonymity we amalgamated features from different assessments and omitted or changed any potentially identifying characteristics and biographical events.

## Results

### Sample Characteristics

Records identified 261 patients, 128 women and 133 men, with a median age of 65 years (32–88) who had been assessed and had completed the HADS. Patients’ characteristics are summarized in Table 1. The HADS classified 88 (33.7%; Table 2) as cases of anxiety, 23 of these also being cases of depression (none reached threshold on the depression scale alone). The HP identified psychological need in 129 patients (49.4%; Table 2).

**Table 1:** Socio-demographic and clinical characteristics of patients assessed by the HP

Variable	Category	*N* = 261	
		*N*	%
Sex	Male	133	51
	Female	128	49
Marital status	Married/living with partner	184	70.5
	Divorced/separated	14	5.4
	Widowed	20	7.7
	Single	24	9.2
	Not recorded	19	7.3
Employment status	Employed	101	38.7
	Homemaker	9	3.4
	Retired	125	47.5
	Unemployed	1	0.4
	Not working because of long-term illness	1	0.4
	Not recorded	24	9.2
Treatment	Enucleation	63	24.1
	Eye conserved		
	Plaque radiotherapy	167	64
	Endoresection	2	0.8
	Endoresection & plaque	14	5.4
	Local resection & plaque	7	2.7
	Tantalum marker insertion before proton beam radiotherapy	7	2.7
	Examination under anaesthetic^a^	1	0.4

^a^Examination of eye for signs of recurrence

**Table 2:** Numbers of patients in whom need for psychological intervention was identified, and whom the HADS classified as cases

Need for psychological intervention	HADS cases & non-cases					
	Anxiety or depression ‘case’			Anxiety or depression ‘non-case’		
	Total	Males	Females	Total	Males	Females
Total	88	33	55	173	100	73
Need identified	70	23	47	59	27	32
Need not identified	18	10	8	114	73	41

### Statistical Analyses

HADS caseness was highly associated with psychological need *χ*^2^ = 48.18, *df* = 1, *p* < .001. Of the 88 patients reaching caseness, the HP identified 70 (79.5%) as having psychological need; in 18 (20.5%), however, she identified no psychological need. Of the 173 patients scoring below the HADS caseness threshold, the HP identified no psychological need in 114 (65.9%), but did identify psychological need in 59 (34.1%). Therefore while, as anticipated, the HADS and the HP’s judgment converged for most patients, they diverged for 77 (29.5%), a substantial minority.

Female gender was associated with both HADS caseness (*χ*^2^ = 9.62, *df* = 1, *p* < .01) and the HP’s judgment of psychological need (*χ*^2^ = 15.19, *df* = 1, *p* < .001), but age and treatment were unrelated to both. In the log linear analysis, 3-way and higher order effects were not significant (χ^2^ = 4.91, *df* = 5, *p* = .43), indicating that whether HADS screening and the HP’s judgment converged or diverged was unrelated to patients’ demographic and clinical characteristics.

### Case Vignettes

Boxes 1, 2, 3, 4, 5 and 6 illustrate patients for whom HADS ‘caseness’ and the HP’s judgment converged or diverged.

**Box 1:** (P1) A ‘true negative’: HADS non-case in whom the HP identified no psychological need (in this and subsequent boxes, case examples are summarized according to the essential elements of the ‘stress and coping’ model; Lazarus & Folkman, 1984)

Male, 65–75 years
**HADS**: Anxiety = 3; Depression = 1 **Personal significance of cancer diagnosis**: P1 felt that diagnosis would have little effect on his life, although he was concerned about how any loss of vision might affect activities such as driving and golf **Coping**: Minimized the seriousness of his diagnosis and wanted to ‘get back to normal’. Doctors had found it early so ‘it would be fine’ **Personal & psychological resources**: Typically approaches difficulties by playing them down to maintain normality; readily accepts offers of practical help **Support**: Large extended circle of family and friends ready to offer practical help P1 maintained that the diagnosis was just ‘one of those things’ that he would deal with it by resuming his normal life as soon, and as best, he could. He had a pragmatic approach to potential loss of vision. The HP judged that the low HADS scores were in line with his ways of coping; that is, by minimizing the diagnosis while focusing realistically on resuming familiar activities after treatment, and in light of his support network, he should be expected to adjust without formal intervention

**Box 2:** (P2) A ‘true positive’: HADS case in whom the HP identified psychological need

Female, 35–45 years
**HADS**: Anxiety = 19; Depression = 10 **Personal significance of cancer diagnosis**: Confirms long-held belief that ‘bad things’ happen to her **Coping**: Worrying, catastrophic thinking; threat monitoring; withdrawal from family **Personal & psychological resources**: Recent sudden bereavement and pre-existing depression leave her ill equipped to cope with the cancer diagnosis **Social Support**: Close family P2 asked to speak with the HP before treatment. Consistent with the HADS score, the HP saw that P2 was extremely upset by her diagnosis which, she felt, confirmed her belief that fate was against her. The HP was concerned that P2 described trying to withdraw emotionally from her young children because ‘I may not be around.’ Against a background of long-standing anxiety, profound grief and low mood, the HP considered that her ways of coping risked compounding her distress and that she therefore needed psychological intervention

**Box 3:** (P3) A ‘true positive’: HADS case in whom the HP identified psychological need

Female, 35–45 years
**HADS**: Anxiety = 16; Depression = 10 **Personal significance of cancer diagnosis**: Challenged her view of herself as a strong, capable and optimistic person and her expectations of being able to continue in her family and work roles, particularly raising her young children into adulthood **Coping**: Worrying about the future; organizing her finances to make her death easier for her family **Personal & psychological resources**: In response to previous crises, she had relied on a sense of optimism, linked to her religious belief, and a confidence in her own strength, but each of these beliefs had been greatly weakened by her diagnosis **Social Support**: Did not feel supported by partner; felt she had no-one else to share her feelings P2 initially presented to the HP assertively as a strong person who was coping well. However, as the HP prompted her to talk more, she described feeling uncharacteristically pessimistic. She disclosed that her attempts to be optimistic were failing and that she was questioning her religious beliefs. She told the HP that she was very anxious about her family should she die. The HP judged that her familiar coping strategies were proving ineffective and that she lacked effective alternative ways of coping and had little available emotional support. She was therefore judged at risk of persistent distress and needed the HP’s help

**Box 4:** (P4) A ‘false positive’: HADS case in whom the HP did not identify psychological need

Male, 55–65 years
**HADS**: Anxiety = 12; Depression = 7 **Personal significance of cancer diagnosis**: Frightened by potential loss of vision and consequent challenge to ability to work and provide for family **Coping**: Information seeking; distraction; social comparison (with other people who were “worse off”); keeping ‘positive’ and not ‘dwelling’ on his problems **Personal & psychological resources**: Has been resilient in the face of previous serious illnesses, and is drawing on ability to frame adverse events positively. He is confident in his ability to apply the same approach to this current diagnosis **Social support**: Supportive network of family and friends Although P4 was shocked and frightened by his diagnosis but, despite the elevated HADS scores, the HP was reassured by his continued ability to use ways of coping which had served him well previously and conferred resilience which was likely to protect him from persistent distress. The HP judged it important not to risk undermining his own way of coping, and therefore simply explained that support was available, should he want it

**Box 5:** (P5) A ‘false negative’: HADS non-case in whom the HP identified psychological need

Male, 65–75 years
**HADS**: Anxiety = 6; Depression = 4 **Personal significance of cancer diagnosis**: Knew he was at risk of liver metastases. A close friend had recently died of liver cancer and he feared for his family’s loss of his ‘guiding role’ were he to develop incurable metastases **Coping**: Internet searching about his condition and treatment; asking others’ opinions; worrying about dying from cancer and about how his family will cope **Personal & psychological resources**: Likes to seek information and deliberate about decisions **Social support**: Close to partner and wider family, but sees himself as the support for them rather *vice versa* P5 had been offered a prognostic biopsy to estimate risk of liver metastases. One family member was strongly advising him against it but, in searching for information beyond what he had been told by the clinical team, and in trying to weigh the advantages and disadvantages, P5 felt in a cycle of uncertainty and Internet searching that left him more confused and undecided. Despite not expressing overt distress to the HP, she judged that his habitual ways of approaching decisions were proving counter-productive. Drawing on health psychology and other literature on decision-making, she suggested that she help guide him through his deliberation. P5 enthusiastically accepted

**Box 6:** (P6) A ‘false negative’: HADS non-case in whom the HP identified psychological need

Female, 60–70 years
**HADS**: Anxiety = 4; Depression = 3 **Personal significance of cancer diagnosis**: Challenged her view of herself as someone her family could depend on in adversity. Exposed her vulnerability to ill health as she aged **Coping**: Worrying **Personal & psychological.resources**: Had typically responded to family and other difficulties by focusing on others’ needs and seeking to be a dependable, resourceful support for them **Support**: Did not feel she could ‘burden’ her family with her own feelings P6 worried about her daughter who was herself experiencing a life-changing illness. Now anticipating her own ageing and decline, P6 feared being unable to support her daughter in future when she most needed her. The HP judged that, whilst not overtly distressed, P6 warranted help in negotiating her life transitions in light of the cancer diagnosis. P6 was positive when offered this help, which provided opportunity for emotional expression and normalization of her accepting support from others

Most patients scored below threshold for’ caseness’ and, in most of these, the HP identified no psychological need. It seemed that these patients successfully ‘disavowed’ the significance of their cancer diagnosis (Salander & Windahl, 1999). For example, P1’s (Box 1) account to the HP showed how he ‘minimized’ the diagnosis, focusing instead on getting on with his life. In those patients who reached ‘caseness’ and whom the HP judged as needing psychological intervention, there were diverse reasons for her judgment. For many patients, given their recent diagnosis, distress was understandable and linked to concerns about cancer recurrence and implications for family. In several, however, as P2 illustrates (Box 2), the HP identified that distress predated diagnosis, being linked to long-standing emotional problems or recent life events. In other patients, she was able to link patients’ distress to contemporaneous stressors, including family responsibilities or relationship problems not directly related to their own diagnosis. Although most patients who reached HADS ‘caseness’ and whom the HP assessed as needing psychological intervention wanted further support, a few did not initially see its potential value, only doing so after discussion with the HP. P3 (Box 3), for example, came to realize that ways of coping that had seen her through previous challenges were insufficient for her cancer diagnosis and that support she had previously relied upon would not be available. Twelve further patients steadfastly declined the HP’s offer of support. In such cases it is important that the HP ensures that patients know how to contact her should their needs, or attitude to having help, change.

For those reaching ‘caseness’ on the HADS, the HP assessed several as not needing psychological intervention because they were experiencing natural emotional reactions to the shock of diagnosis which she judged they could manage without professional intervention. For example, based on eliciting P4’s (Box 4) account of how he coped with previous ill health, the HP agreed with his own appraisal that he could manage his new diagnosis without help. That is, the HP assessed these patients as having resources that would allow them to adjust and recover over time, and she did not wish to undermine this natural process.

Of the patients whom the HADS did not identify as reaching caseness, the HP judged most as not needing psychological intervention because she identified social support, appraisals and ways of coping that helped protect these patients and that equipped them for the future. The minority whom she assessed as having psychological needs showed diverse reasons for this judgment. They had needs that were clearly psychological, although not manifesting as overt distress. For instance, P5 (Box 5) was finding it difficult to decide whether to have a prognostic biopsy. Discussion with the HP showed that his usual approach to difficult decisions was proving problematic. Internet searching had increased his uncertainty and seeking others’ opinions left him susceptible to the strong views of a family member. The HP judged that she could help him by drawing on recent theory about what constitutes a ‘good’ health-care decision (Elwyn & Miron-Shatz, 2009; Salmon & Brown, 2018) and he enthusiastically accepted. A few patients described psychological needs around life transitions associated with their cancer. For instance, through discussion with the HP, P6 (Box 6) came to appreciate how her reaction to the cancer diagnosis had challenged her view of herself as a ‘rock’ of support for her adult family. The HP helped her become able to accept her new feeling of vulnerability by having support from the HP.

## Discussion

For most patients, the HADS score and the HPs’ assessment were aligned. However, they were misaligned in nearly 30% of patients, and these cases have important lessons for HPs and others involved in psychological assessment and support in a cancer context.

### Emotional Distress Does Not Necessarily Indicate a Need for Psychological Intervention

In our clinical context, much of the distress that screening identifies is a natural reaction to extraordinary circumstances. This acute distress reaction is transient in most people (Brennan, 2004; Gao, Bennett, Stark, Murray, & Higginson, 2010; Helgeson, Snyder, & Seltman, 2004), and the HP needs to avoid interfering with the natural adjustment processes that limit this reaction (Dekker et al., 2017). Consistent with this view, many patients distressed by a cancer diagnosis prefer to rely on their own resources in the period immediately after diagnosis, becoming open to psychological intervention only later (Baker et al., 2013). Even then, distress does not necessarily equate to psychological need (Merckaert et al., 2010; Söllner, Maislinger, König, DeVries, & Lukas, 2004). The HP therefore must make careful clinical judgments about the level of involvement to offer. She must decide whether her intervention risks undermining the natural coping of patients, such as P4, who have the resources to manage without her help, or whether it could help patients, such as P2, who seem to lack those.

Patients diagnosed with cancer commonly use emotion-focused coping strategies such as social comparison (Bennenbroek, Buunk, van der Zee, & Grol, 2002), thinking ‘positively’ (Wilkinson & Kitzinger, 2000) and minimizing the seriousness of the diagnosis, as P1 illustrated. Salander and Windahl (1999) described these as examples of ‘disavowal’ whereby, without denying the reality of the diagnosis, patients interpret it in a way that feels more manageable. Similarly, a long-standing view in health psychology is that positive thinking in patients with cancer can help to reduce emotional distress (Dunkel-Schetter, Feinstein, Taylor, & Falke, 1992; Osowiecki & Compas, 1998). However, an opposing view is that, by suppressing emotional expression, emotion-focused coping of this kind increases vulnerability to future distress (Brennan, 2004; Stanton et al., 2000). Given this conflicting evidence, the HP must decide whether patients’ coping is likely to be protective or potentially harmful.

Predictions about the future therefore underlie the HP’s assessment of patients’ needs. That is, she must make clinical judgments based on her assessment of how patients are likely to adjust and cope, with or without intervention. There is some evidence to help her: risk factors for poor adjustment include poor social support (Nordin, Berglund, Glimelius, & Sjödén, 2001), anxiety and depression around the time of diagnosis, personality traits predisposing patients to anxiety and a history of pre-morbid psychological problems (Cook, Salmon, Hayes, Byrne, & Fisher, 2018). Consistent with previous evidence, distress in the present study was unrelated to whether treatment had cost patients the affected eye (Hope-Stone, Brown, Heimann, Damato, & Salmon, 2016). Our finding that women were more likely than men to be distressed, and to be judged as needing psychological intervention, reflects previous findings in studies of cancer generally and uveal melanoma specifically although, contrary to previous evidence, there was no evidence that younger people were more likely to be distressed (Hope-Stone et al., 2016; Linden,Vodermaier, Mackenzie, & Grieg, 2012; Merckaert et al., 2010). In general, the evidence about predictors of distress therefore remains too limited to provide an algorithm that could be used in clinical practice. Therefore, the HP must make a clinical judgment that reflects, not just the broad features of that evidence, but patients’ specific characteristics and circumstances. The approach we have described here, drawing on the stress and coping framework, allows the HP to derive predictions based on a way of formulating patients’ needs and resources that is rooted in health psychology theory. Of course, predictions in this sense are not fixed; they are guides to action for the HP, whose continuing dialogue with the patient allows her continually to revise her formulation and the predictions that result from it.

### Patients Can Have Psychological Needs That are Not Manifest as Emotional Distress

In our survey, the HP judged that further intervention might help more than a third of the patients who were not formally distressed on the HADS. That is, her assessment identified needs that, although psychological, were not manifest in a ‘clinical’ level of distress. Of course, any screening instrument has some degree of unreliability, so some of these patients might have been clinically distressed. However, in cancer care generally, patients’ distress measured on a screening instrument correlates only poorly or not at all with their desire for formal psychological support (Merckaert et al., 2010; Söllner et al., 2004). It seems, therefore, that patients seek psychological help for reasons that are not necessarily manifest as distress detectable on the HADS. Salander (2010) described some of these reasons when he audited referrals to a psycho-oncology service; diagnosis of cancer can expose pre-existing inter-personal problems, or it can bring challenges in adjusting to a changed life situation.

Patients in our survey also needed to make important decisions about medical intervention options. Given the evidence (Cook et al., 2011; Peters, McCaul, Stefanek, & Nelson, 2006) that patients typically make decisions using heuristic ‘short-cuts’ that might not reflect their best interests, the HP can provide psychologically informed guidance, for example by helping them anticipate potential outcomes and how they are likely to respond (Elwyn & Miron-Shatz, 2010). The challenge for the HP is, therefore, to recognize and respond to needs that are ‘psychological’ but do not present as overt emotional distress.

### Psychologists Need to Escape the Medical Model of Psychological Need

Equating need with distress reflects the persistence of a medical, ‘diagnostic’ model of psychological need. By contrast, Salmon et al. (2015) argued for a broader, ‘public health’ framework which recognizes that need can be identified in different ways: ‘normative need’ identified by experts; ‘comparative need’, where a criterion for need that is established in one population is used in another; ‘felt need’, when patients want help; and ‘expressed need’ when they seek help. The use of a questionnaire such as the HADS, which is validated against psychiatrists’ judgments, reflects only normative and comparative need. Coyne (2013b) argued that screening for distress could rely, instead, on patients’ own views of their needs—i.e. felt and expressed need. However, such reliance risks denying some patients help that they might benefit from; patients can be unaware of what professional support can offer, or they might reject it because they think that they do not warrant help. Moreover, patients’ initial assertions that they can manage without help might well be expressions of psychological need, as P3 (Box 3) illustrated. Some patients in our study only accepted the HP’s help after discussion in which the HP could explain its purpose and nature. Therefore, the HP’s responsibility to judge psychological need can no more be delegated to patients by asking whether or not they want help than it can be based solely on a HADS score. Whether need exists in any patient reflects a weighing of the different criteria for need. Where they align, need is generally unambiguous. Where they do not, the HP must make a clinical judgment.

## Conclusion: Dialogue with the Patient as the Vehicle for Psychological Assessment

In our service, the HP’s assessment is based on dialogue with the patient, informed and structured by health psychology theory. Compared to this, a questionnaire, which is widely used to screen for psychological need in cancer services, often proved misleading. Similarly, Bonacchi et al. (2010) found that using clinical interviews allowed psychologists to identify psychological need in more patients than those who, based on questionnaire scores, would have met criteria for distress. Our observations therefore reverse the conclusion of previous reports that, compared to formal screening questionnaires, practitioners’ judgments of patients’ psychological needs are often inaccurate (Mitchell, Hussain, Grainger, & Symonds, 2011; Singer et al., 2011). Those reports, in effect, regarded the screening questionnaire as a ‘gold standard’, disregarding the inevitably imperfect reliability of questionnaires and excluding the kinds of considerations that contributed to the HP’s assessment in our unit. Whilst a screening tool can be informative, we argue, consistent with Bonacchi et al.’s (2010) advice, that dialogue with patients is essential for a professional judgment of whether psychological need exists, and whether psychological intervention is warranted.

By contrast, current health policy tries to simplify identification of psychological need in cancer care so that it does not require a psychologist, by using formal measures of distress to indicate need. Even if the non-psychological practitioners administering such screening instruments were to look beyond the formal result of screening, it is not clear to what extent their judgments could reflect the kinds of considerations that shape those of a psychological practitioner such as the HP, who is psychologically trained and supervised, and whose judgments are informed by psychological theory. Currently in UK cancer care, many nurses are now trained in Holistic Needs Assessment (HNA) which comprises a ‘concerns thermometer’ and problem checklist to assess patients’ current sources of concern, including distress but also physical symptoms and practical problems (Young, Cund, Renshaw, Quigley, & Snowden, 2015). While guidance around the HNA emphasizes the importance of dialogue, it is confined to specific concerns that patients are able to express, i.e. ‘expressed need’ in the public health framework, above. We acknowledge that it may be the best that services without a HP can do, but it is unlikely to substitute for expert psychological assessment. Nevertheless, clinical staff involved in screening will need to be alert to the limitations of screening. They need to avoid withholding help from patients who require it but do not manifest distress on a screening instrument or, conversely, intervening when distress signifies a normal process of adjustment. Future research, training and evaluation will need to examine how clinical staff can depart from screening algorithms and use dialogue with the patient to assess for psychological need. Of course, screening for distress is only effective to the extent that a service can address any needs that are identified. Having a psychologist in a service provides for addressing needs as well as identifying them. Indeed, the dialogue of the assessment itself starts the process of intervention.

## References

[CR1] Baker P, Beesley H, Dinwoodie R, Fletcher I, Ablett J, Holcombe C, Salmon P (2013). You’re putting thoughts into my head: A qualitative study of the readiness of patients with breast, lung or prostate cancer to address emotional needs through the first 18 months after diagnosis. Psycho-Oncology.

[CR2] Bennenbroek FTC, Buunk BP, van der Zee KI, Grol B (2002). Social comparison and patient information: What do cancer patients want?. Patient Education and Counseling.

[CR3] Bonacchi A, Rossi A, Bellotti L, Franco S, Toccafondi A, Miccinesi G, Rosselli M (2010). Assessment of psychological distress in cancer patients: A pivotal role for clinical interview. Psycho-Oncology.

[CR4] Brennan J (2004). Cancer in context a practical guide to supportive care.

[CR5] Brennan J (2012). Refinement of the distress management problem list as the basis for a holistic therapeutic conversation among UK cancer patients with cancer. Psycho-Oncology.

[CR6] Cook S, Damato B, Salmon P (2011). Reconciling the principle of patient autonomy with the practice of informed consent: Decision making about prognostication in uveal melanoma. Health Expectations.

[CR7] Cook S, Salmon P, Hayes G, Byrne A, Fisher PL (2018). Predictors of emotional distress a year or more after diagnosis of cancer: A systematic review of the literature. Psycho-Oncology.

[CR8] Coyne JC (2013). Second thoughts about implementing routine screening of cancer patients for distress. Psycho-Oncology.

[CR9] Coyne JC (2013). Benefits of screening cancer patients for distress still not demonstrated. British Journal of Cancer.

[CR10] Damato B (2001). Detection of uveal melanoma by optometrists in the United Kingdom. Ophthalmic and Physiological Optics.

[CR11] Damato B (2010). Does ocular treatment of uveal melanoma influence survival?. British Journal of Cancer.

[CR14] Dekker J, Braamse A, Schuurhuizen C, Beekman AT, van Linde M, Sprangers MA, Verheul HM (2017). Distress in patients with cancer–on the need to distinguish between adaptive and maladaptive emotional responses. Acta Oncologica.

[CR16] Deshields TL, Nanna SK (2010). Providing care for the “whole patient” in the cancer setting: The psycho oncology consultation model of patient care. Journal of Clinical Psychology in Medical Settings.

[CR17] Dunkel-Schetter C, Feinstein LG, Taylor SE, Falke RL (1992). Patterns of coping with cancer. Health Psychology.

[CR18] Elwyn G, Miron-Shatz T (2010). Deliberation before determination: The definition and evaluation of good decision making. Health Expectations.

[CR20] Gao W, Bennett MI, Stark D, Murray S, Higginson IJ (2010). Psychological distress in cancer from survivorship to end of life care: Prevalence, associated factors and clinical implications. European Journal of Cancer.

[CR21] Helgeson VS, Snyder P, Seltman H (2004). Psychological and physical adjustment to breast cancer over 4 years: Identifying distinct trajectories of change. Health Psychology.

[CR22] Hope-Stone L, Brown S, Heimann H, Damato B, Salmon P (2016). Two-year patient- reported outcomes following treatment for uveal melanoma. Eye.

[CR23] Kujala E, Mäkitie T, Kivelä T (2003). Very long-term prognosis of patients with malignant uveal melanoma. Investigative Ophthalmology & Visual Science.

[CR24] Lazarus RS, Folkman S (1984). Stress appraisal and coping.

[CR25] Lilliehorn S, Hamberg K, Kero A, Salander P (2010). ‘Admission into a helping plan’: A watershed between positive and negative experiences in breast cancer. Psycho-Oncology.

[CR26] Linden W, Vodermaier A, MacKenzie R, Grieg D (2012). Anxiety and depression after cancer diagnosis: Prevalence rates by cancer type, gender and age. Journal of Affective Disorders.

[CR27] Litz BT, Gray MJ, Bryant RA, Adler AB (2002). Early intervention for trauma: Current status and future directions. Clinical Psychology: Science and Practice.

[CR28] Merckaert I, Libert Y, Messin S, Milani M, Slachmuylder Jean-Louis, Razavi D (2010). Cancer patients desire for psychological support: Prevalence and implications for screening patients’ psychological needs. Psycho-Oncology.

[CR29] Mitchell AJ, Hussain N, Grainger L, Symonds P (2011). Identification of patient-reported distress by clinical nurse specialists in routine oncology practice: A multicentre UK study. Psycho-Oncology.

[CR30] Nordin K, Berglund G, Glimelius B, Sjödén PO (2001). Predicting anxiety and depression among cancer patients: A clinical model. European Journal of Cancer.

[CR31] Osowiecki D, Compas BE (1998). Psychological adjustment to cancer: Control beliefs and coping in adult cancer patients. Cognitive Therapy and Research.

[CR32] Peters E, McCaul KD, Stefanek M, Nelson W (2006). A heuristics approach to understanding cancer risk perception: Contributions from judgment and decision-making research. Annals of Behavioral Medicine.

[CR33] Salander P (2010). Motives that cancer patients in oncological care have for consulting a psychologist. Psycho-Oncology.

[CR34] Salander P, Windahl G (1999). Does ‘denial’ really cover our everyday experiences in clinical oncology? A critical view from a psychoanalytic perspective on the use of ‘denial’. The British Journal of Medical, Psychology.

[CR35] Salmon, P., & Brown, S. (2018) Reconciling the theory and reality of shared decision-making: A ‘matching’ approach to practitioner leadership. *Health Expectations*, under revision. 10.1111/hex.12853 PMC6543140 30478979

[CR36] Salmon P, Clark L, McGrath E, Fisher P (2015). Screening for psychological distress in cancer: Renewing the research agenda. Psycho-Oncology.

[CR37] Singer S, Brown A, Einenkel J, Hauss J, Hinz A, Klein A, Brähler E (2011). Identifying tumor patients’ depression. Supportive Care in Cancer.

[CR38] Söllner W, Maislinger S, König A, DeVries A, Lukas P (2004). Providing psychosocial support for breast cancer patients based on screening for distress within a consultationliaison service. Psycho Oncology.

[CR39] Stanton AL, Danoff-Burg S, Cameron CL, Bishop M, Collins CA, Kirk SB, Twillman R (2000). Emotionally expressive coping predicts psychological and physical adjustment to breast cancer. Journal of Consulting and Clinical Psychology.

[CR40] Vodermaier A, Millman R (2011). Accuracy of the Hospital Anxiety and Depression Scale as a screening tool in cancer patients: A systematic review and meta-analysis. Supportive Care in Cancer.

[CR41] Watson M, White C, Davolls S, Mohammed A, Lynch A, Mohammed K (2013). Problemfocussed interactive telephone therapy for cancer patients: A phase II feasibility trial. PsychoOncology.

[CR42] Watson M, White C, Lynch A, Mohammed K (2017). Telephonedelivered individual cognitive behavioural therapy for cancer patients: An equivalence randomised trial. PsychoOncology.

[CR43] Wilkinson S, Kitzinger C (2000). Thinking differently about thinking positive: A discursive approach to cancer patients’ talk. Social Science & Medicine.

[CR44] Young J, Cund A, Renshaw M, Quigley A, Snowden A (2015). Improving the care of cancer patients: Holistic needs assessment. British Journal of Nursing.

[CR45] Zigmond AS, Snaith RP (1983). The hospital anxiety and depression scale. Acta Psychiatrica Scandinavica.

